# Protein C and protein S deficiencies may be related to survival among hemodialysis patients

**DOI:** 10.1186/s12882-019-1344-8

**Published:** 2019-05-28

**Authors:** Mayuri Ichinose, Naru Sasagawa, Tetsuo Chiba, Katsuhide Toyama, Yuzo Kayamori, Dongchon Kang

**Affiliations:** 1 Division of Nephrology and Hypertension, Department of Internal Medicine, The Jikei University School of Medicine, 3-25-8, Nishi-Shimbashi, Minato-ku, Tokyo, 105-8461 Japan; 2 Vascular Access Center, Yokohama Dai-ichi Hospital, Yokohama, Japan; 3 Department of Internal Medicine, Yokohama Dai-ichi Hospital, Yokohama, Japan; 4 Department of Health Sciences, Faculty of Medical Sciences, Kyushu University, Fukuoka, Japan; 5 Department of Clinical Chemistry and Laboratory Medicine, Graduate School of Medical Sciences, Kyushu University, Fukuoka, Japan

**Keywords:** Hemodialysis patients, Protein C, Protein S, Thrombophilia

## Abstract

**Background:**

Thrombophilia due to protein C (PC) and protein S (PS) deficiencies is highly prevalent among patients with stage 5 chronic kidney disease and is reported to arise due to extracorporeal circulation during hemodialysis (HD). This study aimed to evaluate the relationship between HD treatment and thrombophilia.

**Methods:**

A total of 114 Japanese patients on maintenance HD (62 men, 52 women) were followed during 2008–2011. Their survival rates were compared against the duration of HD. Prior to each HD, coagulation/fibrinolysis parameters and PC and PS activities were measured using standard techniques. The patients were divided into two groups: Group 1, with PC and/or PS deficiencies (*n* = 32), and Group 2, without PC and PS deficiencies (*n* = 82). The influence of such deficiencies and duration of dialysis on survival was examined. Time-to-event analysis was applied using Kaplan-Meier estimates, and the log-rank test was proposed to test the equivalence of relative survival data. Hazard ratios and 95% confidence intervals (CI) were calculated.

**Results:**

Of the 114 patients, 37 died (Group 1, 22; Group 2, 15). The hazard ratio (95% CI) was higher (*p* = 0.004) in Group 1 than Group 2. Gene analyses of PC and PS were performed in 14 patients from Group 1. No mutations in either protein were observed. We analyzed the causes of death in both groups; however, the estimated thrombophilia-related incidence of death could not be determined due to small sample size of HD patients.

**Conclusions:**

Our results suggest that PC and PS deficiencies may be related to survival in HD patients. However, this finding warrants additional research.

## Background

Chronic kidney disease (CKD) is a global health burden affecting 7% of people aged > 30 years and 25–35% of people aged > 65 years [1]. In patients who require hemodialysis (HD), the glomerular filtration rate is reduced to 10–15% [2]. Several studies have revealed that HD patients are at high risk of cardiovascular disease (CVD) [3]. CVD incidence is significantly higher among HD patients, with mortality rates 10–20 times greater than those of the general population [1]. Patients on maintenance HD experience thrombotic events that frequently occur at the site of vascular access as well as in the coronary, cerebral, and retinal arteries [4, 5]. Thrombosis-related cardiovascular events are well-known dominant causes of death and account for the majority of morbidities in end-stage renal disease (ESRD) patients [6]. Cardiovascular events have several risk factors, including lipoproteinemia, chronic inflammation, hypertension, oxidative stress, elevated homocysteine level, and anemia. These factors may contribute to the increased frequency of critical complications in CKD patients [7–9].

HD is a very specific procedure that involves the extracorporeal removal of excess fluid and waste products, including uremic toxins and creatinine, from the blood. CKD patients, especially those on HD, have a hypercoagulable state [10, 11]. HD-associated thrombosis occurs frequently in the clinical field, and the association between HD and thrombosis could be a causal relationship [11]; however, the mechanism remains unclear. Thrombosis involves various genetic and environmental factors [12, 13]. Under normal physiological conditions, the mechanism of coagulation control works in a multilevel cascade to prevent clot formation in blood vessels, and coagulation reactions do not occur excessively even if the blood vessels are injured. In HD, the blood circuit resembles a condition of artificially injured vessels, and some studies report that deficiency in the coagulation inhibition of protein C (PC) and protein S (PS), which are regulators of physiological coagulation, poses a risk for thrombus formation [12, 13]. It is well known that there are many more dialysis patients with a history of long-term HD in Japan compared to those in European countries and the United States. While preparing for this study, our preliminary findings seem to suggest an abnormal association between PC and PS activities and duration of HD and that long-term HD patients have normal or high PC and PS activity levels. Therefore, in this study, we aimed to evaluate PC and PS activity in HD patients retrospectively; we also assessed the patients over a 3-year follow-up period to clarify how this association affected their survival.

## Methods

A total of 114 patients on HD were enrolled in this study and followed up from October 2008 to December 2011. Their characteristics, including the cause of ESRD and duration of hemodialysis, are shown in Table 1. All patients were dialyzed using primary arteriovenous fistulas (AVFs); there were no arteriovenous grafts (AVGs) and artificial devices. No patients were taking drugs known to interact with PC and PS activities such as warfarin, at the starting point. All patients received conventional HD with bicarbonate bath thrice a week and underwent dialysis using a polysulfone membrane when they were enrolled. Heparin was used as an anticoagulant in all patients. Information was obtained from all patients about the family history of thrombotic related diseases, history of cerebral infarction, and also their social history such as smoking, drinking, or medications other than those related to HD.

**Table 1:** Basic distribution of PC and PS activities in study patients compared with duration of HD

Protein C activity %					
Duration of HD(years)	0~29	30~70	70~100	100~	
1~5	1	18	22	16	57
6~10	2	6	15	11	34
11~15		2	3	2	7
16~20			6	4	10
21~25			1	4	5
26~				1	1
Total number of patients	3	26	47	38	114
Protein S activity %					
Duration of HD(years)	0–29	30–70	70–100	100-	
1~5	1	19	28	8	56
6~10	1	8	17	10	36
11~15		1	3	2	6
15~20		1	6	3	10
21~25			2	3	5
26~			1		1
Total number of patients	2	29	57	26	114

The study protocol was verified and approved by the institutional review board of Kyushu University. Written informed consent was obtained from all patients, and all clinical investigations were conducted according to the principles laid down in the Declaration of Helsinki. The patients gave consent for the publication of clinical details.

### Laboratory investigations

Venous blood samples were collected at the beginning of each week just after the start of HD before heparin injection, and various parameters were measured. Hematology parameters included hematocrit (Ht) and platelet count (Plt). Biochemistry parameters included serum creatinine (Cre), blood urea nitrogen (BUN), serum uric acid (UA), total cholesterol (T-Cho), and triglycerides (TG). Coagulation testing of plasma samples was performed using an automated blood coagulation analyzer. Coagulation parameters included prothrombin time (PT-T), prothrombin time percentage (PT %), prothrombin time - international normalized ratio (PT-INR), thrombo-test (TT), hepaplastin test (HPT), and activated partial thromboplastin time (APTT). PC activity was measured using a commercial enzyme-linked immunosorbent assay (ELISA) kit, whereas PS was measured as described previously [14].

### Statistical analysis

Students’ paired t-test was used for parametric data, and multivariate analyses were performed with logistic analyses to determine the significance of differences between groups. All analyses were performed using SPSS Statistics version 23 (IBM Corp., Armonk, NY, USA). Clinical data were expressed as mean values ± standard deviation and as prevalence rates. Statistical significance was taken at or below the 5% level. Time-to-event analysis was applied using Kaplan-Meier estimates, and the log-rank test was proposed to test the equivalence of relative survival data. Hazard ratios and 95% confidence intervals (CI) were calculated.

## Results

We divided the patients with PC and PS activity into two groups: Group 1 consisted of 32 patients with decreased PC and PS activities, and Group 2 consisted of 82 patients with PC or PS activities either within the normal range or increased.

The causes of renal failure that led to ESRD among the patients are shown in Table 2. In Group 1, the causes included chronic glomerulonephritis (CGN) (25%), diabetes mellitus (DM) (62.5%), polycystic kidney (PCK) (3%), and focal glomerulosclerosis (FGS) (9.4%). In Group 2, the causes were CGN (29.3%), DM (51.2%), PCK (3.7%), and FGS (13.4%).

**Table 2:** Basic demographics and clinical characteristics of study patients

Group 1	*N* = 32	PC and/or PS activities were low	
Group 2	*N* = 82	Both PC and PS activities were normal	
		Group 1	Group 2
No. of cases		32	82
Age		58.4 ± 10.9 y	57.6 ± 10.4 y
Duration of hemodialysis		14 y ± 3 mo	13 y ± 7 mo
Cause of renal failure			
CGN		8	24
DM		20	42
PCK		1	3
FGS		3	11
Others		0	2

*PC* protein C, *PS* protein S, *y* years, *mo* months, *CGN* chronic glomerulonephritis, *DM*, diabetes mellitus, *PCK* polycystic kidney, *FGS* focal glomerulosclerosis

Both groups showed similar levels of blood and biochemical parameters (Ht, Plt, Cre, BUN, UA, T-Cho, and TG) (Table 3), demonstrating no significant differences (*p* = 0.05).

**Table 3:** Blood and biochemical parameters

		Group 1	Group 2	*p*-value
Ht	%	28.6 ± 3.17	28.85 ± 2.86	NS
Plt	× 10^3^	20.56 ± 7.56	21.99 ± 7.45	NS
BUN	mg/dL	72.23 ± 18.49	73.39 ± 15.27	NS
Cre	mg/dL	12.4 ± 1.86	12.57 ± 2.94	NS
UA	mg/dL	8.38 ± 1.29	8.06 ± 1.41	NS
Ca	mg/dL	9.88 ± 0.89	9.92 ± 1.01	NS
Pi	mg/dL	5.87 ± 0.97	5.78 ± 1.26	NS
T-Cho	mg/dL	162.48 ± 34.23	172.63 ± 41.33	NS
TG	mg/dL	155.96 ± 96.25	152.93 ± 91.11	NS

*Ht* hematocrit, *NS* not significant, *Plt* platelet count, *BUN* blood urea nitrogen, *Cre* serum creatinine, *UA* serum uric acid, *Ca* serum Calcium, *Pi* Serum phosphate (inorganic), *T-Cho* total cholesterol, *TG* triglyceride

There were also no significant differences in blood coagulation parameters between the patients in Group 1 and Group 2 who underwent the blood coagulation test (*p* = 0.05) (Table 4).

**Table 4:** Blood coagulation parameters

		Group 1	Group 2	*p*-value
PT-T	sec	12.29 ± 1.05	12.54 ± 2.30	NS
PT	%	87.9 ± 15.88	86.08 ± 16.27	NS
PT-INR		1.07 ± 0.97	1.1 ± 0.21	NS
TT	%	99.09 ± 6.51	96.26 ± 14.94	NS
HPT	%	115.23 ± 11.96	114.45 ± 16.77	NS
APTT	sec	35.76 ± 5.89	37.44 ± 12.2	NS
Fibrinogen	mg/dL	320.23 ± 107.16	296.93 ± 97.24	NS
PC activity	%	38.78 ± 20.29*	97.02 ± 17.71	*< 0.01*
PS activity	%	34.16 ± 26.56*	81.38 ± 19.95	*< 0.01*

*NS* not significant, *PT-T* prothrombin time, *PT%* prothrombin percentage, *PT-INR* prothrombin time - international normalized ratio, *TT* thrombotest, *HPT* hepaplastin test, *ATPP* activated partial thromboplastin time, *PC* protein C, *PS* protein S

*p < 0.01 vs Group 2 (analysis of variance)

In both groups of patients, there was family history of myocardial infarction (MI) (Group 1, 9%; Group 2, 10%); cerebrovascular disease (CVD) (Group 1, 24%; Group 2, 19%); deep vein thrombosis (DVT) (Group 1, 2%; Group 2, 3%); and peripheral artery disease (PAD) (Group 1, 1%; Group 2, 3%). The patients also had past medical history of MI (Group 1, 10%; Group 2, 9%); CVD (Group 1, 8%; Group 2, 7%); DVT (Group 1, 5%; Group 2, 6%), pulmonary embolism (Group 1, 3%; Group 2, 1%); and PAD (Group 1, 3% Group 2, 3%). There was no significant difference between the groups in terms of duration of HD. No obvious relationship was noted with other factors such as smoking, drinking, and medications, between Groups 1 and 2. During the 3-year follow-up, 36 patients died.

**Fig. 1:**
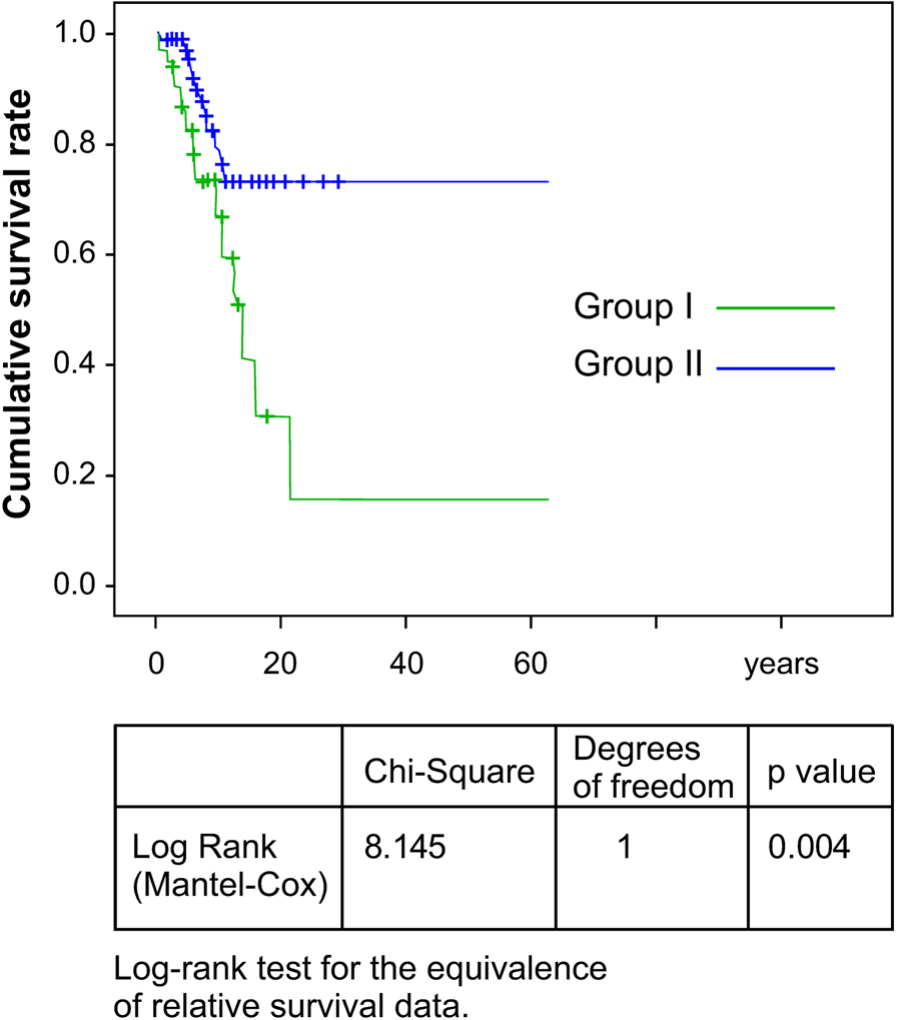
Survival curves calculated using the Kaplan-Meier method according to the duration of analysis

A 3-year follow-up after the blood tests revealed that Group 1 had significantly higher mortality rates than Group 2. The Kaplan-Meier survival analysis showed a significant difference in all-cause mortality between both groups (*p* = 0.004, by the log-rank test) (Fig. 1). Causes of death in the 37 patients included heart failure (Group 1, 8; Group 2, 6); cerebral vascular disease (Group 1, 3; Group 2, 4); pneumonia (Group 1, 3; Group 2, 3); and unknown (Group 1, 8; Group 2, 2).

Gene analyses were performed in 14 patients from Group 1, who provided informed consent and agreement to undergo a gene mutation screening of PC and PS. However, no mutations in either protein were observed.

## Discussion

According to the US Renal Data System and the Die Deutsche Diabetes Dialyse Studie (4D Study), approximately 50% of deaths among HD patients are from cardiovascular causes [15]. However, cardiovascular events could be diverse and related to other factors especially in HD patients. In this study, we observed that PC and/or PS activities decreased in some HD patients (Group 1). HD patients (Group 1) also had significantly higher mortality rates than those with normal PC and PS activities (Group 2) during the 3-year follow-up. Moreover, no PC and PS gene mutations were found in Group 1. One study showed the recovery of PC activity after renal transplantation [16]. This could be explained by the interplay between blood and dialyzer membrane surfaces and the use of anticoagulants in the HD circuit. These factors induce acute and chronic activation of platelets, resulting in platelet exhaustion along with consumption of PC and PS [17]. Furthermore, the duration of HD was longer in Group 1 than in Group 2, which might have led to the decrease in PC and PS activities in Group 1 due to dissipation of these proteins in the HD circuit. A study from Japan showed that the duration of HD in HD patients is a significant independent risk factor for mortality [18].

Patients with chronic renal failure have a high prevalence of systemic inflammation and diffuse endothelial damage [19]. The endothelium modulates the systemic coagulation cascade by inhibiting activated coagulation factors, such as V and VIII, through the PC and PS pathway. The pathway starts when thrombin binds to endothelial cell receptors on the endothelial cell surface [20]. Endothelial injury results in decreased levels of membrane-bound thrombomodulin and increased levels of soluble thrombomodulin in the plasma [21]. This could be attributed to a decrease in PC activity. PC deficiency can occur during acute thrombosis or when the patient is undergoing anticoagulation. However, this possibility is low in the present study because the values of blood coagulation and hematology parameters were similar in both groups. Plasma levels of PC were reported to be significantly lower in HD patients with thrombotic complications than in those without thrombotic complications [22]. Moreover, the levels of anti-PC and anti-PS antibodies were significantly higher in HD patients with vascular access thrombosis than in those without vascular access thrombosis [23].

HD patients were reported to have a family history of CKD or ESRD and a higher risk for developing these conditions [24], and the leading cause of death among HD patients is cardiovascular disease [6]. Additionally, the presence of a residual thrombus after the first episode of DVT is an independent risk factor for recurrence [25]. Stable ischemic heart disease and previous MI are also well known to easily exacerbate the risk of recurrent cardiovascular events [26]. Recently, some studies have evaluated different risk factors for thrombophilia in HD patients [17, 27]. In the present study, both groups of HD patients also reported a high prevalence of CVD and MI in their family and medical histories, and a high morbidity of CVD. Therefore, transfer to another clinic or hospital was required. This prevented us from tracking their medical condition and dialysis activity in detail, and added to the difficulty in analyzing some of the factors. Because dialysis is a treatment by specialists from different fields, further research on HD patients with thrombophilia within a multidisciplinary cooperative system is required.

Our study included only HD patients and therefore the sample size was too small to reveal basic causes of thrombophilia-related death. Additional large-scale research is required, especially among nephritis and chronic renal failure patients, to further clarify the unknown risk factors of thrombophilia among such patients.

## Conclusions

In conclusion, PC and PS deficiencies are not only associated with vascular access thrombosis [28] but may also be related to survival among HD patients. It is important that the PC and PS activities, in addition to thrombophilia, among HD patients be carefully monitored, in particular, by clinicians and experts in hematology and nephrology.

## Abbreviations

APTT: Activated partial thromboplastin time
BUN: Blood urea nitrogen
CKD: Chronic kidney disease
Cre: Creatinine
ELISA: Enzyme-linked immunosorbent assay
HD: Hemodialysis
HPT: Hepaplastin test
Ht: Hematocrit
INR: International normalized ratio
PC: Protein C
Plt: Platelet count
PS: Protein S
PT %: Prothrombin time percentage
PT-T: Prothrombin time
T-Cho: Total cholesterol
TG: Triglycerides
TT: Thrombo-test
UA: Serum uric acid
